# A crowdsourcing workflow for extracting chemical-induced disease relations from free text

**DOI:** 10.1093/database/baw051

**Published:** 2016-04-16

**Authors:** Tong Shu Li, Àlex Bravo, Laura I. Furlong, Benjamin M. Good, Andrew I. Su

**Affiliations:** ^1^Department of Molecular and Experimental Medicine, the Scripps Research Institute, La Jolla, CA 92037, USA; ^2^Research Programme on Biomedical Informatics (GRIB), IMIM, UPF, Barcelona, Spain

## Abstract

Relations between chemicals and diseases are one of the most queried biomedical interactions. Although expert manual curation is the standard method for extracting these relations from the literature, it is expensive and impractical to apply to large numbers of documents, and therefore alternative methods are required. We describe here a crowdsourcing workflow for extracting chemical-induced disease relations from free text as part of the BioCreative V Chemical Disease Relation challenge. Five non-expert workers on the CrowdFlower platform were shown each potential chemical-induced disease relation highlighted in the original source text and asked to make binary judgments about whether the text supported the relation. Worker responses were aggregated through voting, and relations receiving four or more votes were predicted as true. On the official evaluation dataset of 500 PubMed abstracts, the crowd attained a 0.505 *F*-score (0.475 precision, 0.540 recall), with a maximum theoretical recall of 0.751 due to errors with named entity recognition. The total crowdsourcing cost was $1290.67 ($2.58 per abstract) and took a total of 7 h. A qualitative error analysis revealed that 46.66% of sampled errors were due to task limitations and gold standard errors, indicating that performance can still be improved. All code and results are publicly available at https://github.com/SuLab/crowd_cid_relex

**Database URL**: https://github.com/SuLab/crowd_cid_relex

## Introduction

Spurred by advancements in high-throughput analytical techniques like genomic sequencing and microarrays, biology is undergoing a rapid transition to a field requiring large scale data analysis (1, 2). Inexpensive methods for generating plentiful data have enabled precision medicine to emerge as the next frontier of adaptive, responsive and individualized health management and patient care (3, 4). In this new era of biology, predictive modeling and information retrieval now play influential roles in determining experimental success (5). Precision medicine’s need for a clear and comprehensive big picture in the resulting data deluge has in turn driven exploration of systems biology- and literature-based approaches using complex network-based models (6, 7).

The drug discovery field in particular has embraced literature-based approaches in an attempt to increase the probability of drug candidate success in an era of increasingly stringent clinical trials (8–10). A critical component of creating successful drugs involves accurately predicting adverse effects, which are one major reason why drugs fail during clinical trials (11, 12). Mining the existing biomedical literature for chemical–disease relations (CDRs) is central to being able to accurately predict adverse effects, and therefore of great interest to pharmaceutical companies (13).

Although well-known manual curation efforts like the Comparative Toxicogenomics Database have already curated over a hundred thousand documents for CDRs, alternative methods are needed, since expert manual biocuration would be impractically expensive to apply to the entire literature (14). Researchers have therefore turned to automated CDR extractors to address issues of scalability. Knowledge-based (15) and machine learning-based (16) methods have been applied to PubMed abstracts and medical case reports (17) with varying levels of success. However, difficulties with named entity recognition (NER) and anaphora resolution, common among many biological natural language processing (NLP) methods, have hindered progress (18–20).

We sought to improve upon current CDR extraction methods and circumvent the experienced difficulties through crowdsourcing. Crowdsourcing, a collection of approaches involving outsourcing tasks to members of the public, has gained traction in recent years in the NLP domain for its ability to quickly and cheaply gather large numbers of independent human judgments (21). It is used to generate gold standard data for training machine learning systems (22) and as a way to directly enhance the efficiency of annotation pipelines that involve manual labor (23–25). Although Burger *et al.* (23) extracted gene-mutation relations using crowdsourcing, and Khare *et al.* (24) tackled disease indications from drug labels, crowdsourcing has not yet been applied to annotating PubMed abstracts for CDRs.

Here we describe a crowdsourcing approach to extracting CDRs from PubMed abstracts in the context of the BioCreative V community-wide biomedical text mining challenge (26, 27), and provide an assessment of its efficiency and accuracy as compared with the expert-generated gold standard.

## Materials and Methods

**Figure 1.**
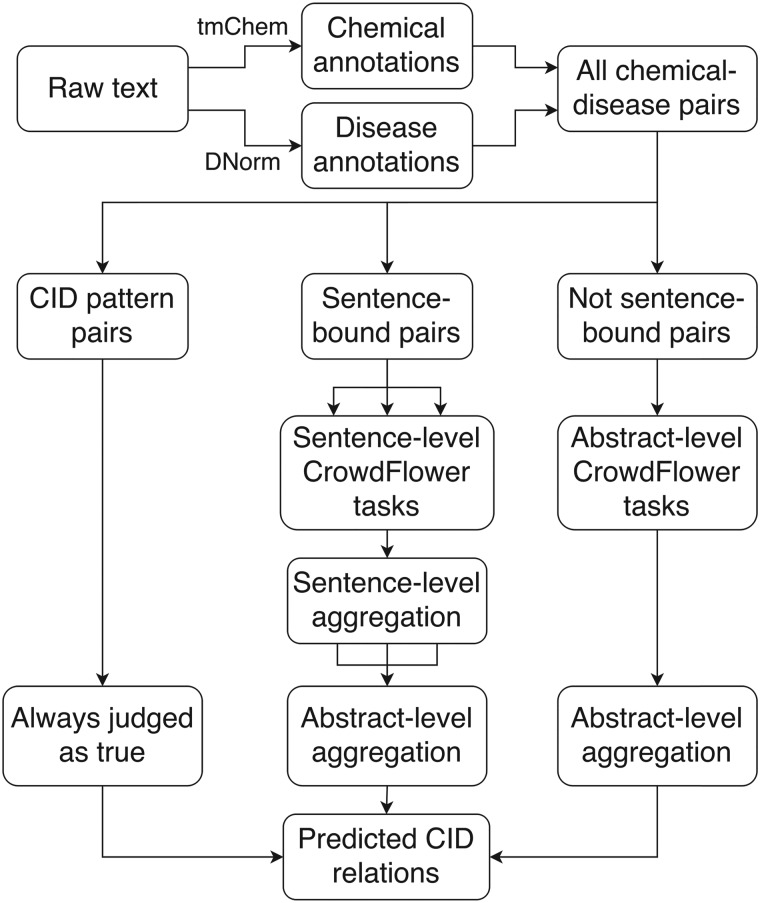
Crowdsourcing workflow for extracting CID relations from free text. DNorm and tmChem were used to annotate disease and chemical concepts in the text. All possible pairwise combinations of chemicals and diseases were generated and processed either automatically or via a sentence- or abstract-scoped crowdsourcing task using the CrowdFlower interface.

Of the two subtasks for the CDR challenge, we focused our crowdsourcing approach exclusively on the relation extraction subtask. We used the provided tools of tmChem (28) and DNorm (29) to perform chemical and disease NER, respectively, and processed potential chemical-induced disease (CID) relations either automatically or with one of two crowdsourcing workflows (Figure 1).

First, we used tmChem and DNorm to generate a set of Medical Subject Heading (MeSH) annotations from the provided raw text. To boost NER performance, we resolved acronyms without attached MeSH identifiers by matching them to other identified annotations using a rule-based pattern (Supplementary Material 1). With this rule, examples like the six instances of ‘BPA’ from PMID 23871786 (‘mice following BPA exposure … 50 mg BPA/kg diet … pubertal BPA exposure’) were resolved to the MeSH ID for ‘bisphenol A’. We found that NER performance on an annotation level for chemicals increased from 0.814 to 0.854 *F*-score after resolving acronyms (Supplementary Material 1). Disease NER performance did not change.

Next, all unique chemical–disease MeSH identifier pairs were generated from the set of annotations. Concepts missing MeSH identifiers or using identifiers from other ontologies (e.g. Chemical Entities of Biological Interest (CHEBI)) were ignored. This set of all possible potential relations was divided into three mutually exclusive classes. Relation pairs which matched a simple CID pattern (chemical annotation occurred no >15 characters before disease annotation, and the text between them contains ‘induce’) were filtered automatically and judged to be true, and were never shown to a crowd. This pattern was chosen because earlier work showed that it was the pattern most frequently used to describe drug side effects in FDA drug labels (16).

**Figure 2.**
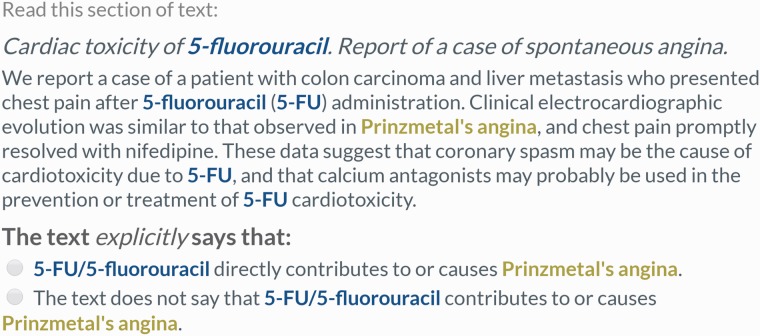
Example of an abstract-scoped relation extraction task. Workers were shown the entire abstract when none of the annotations of the chemical–disease pair ever co-occurred within the same sentence. Workers were asked to make a single choice about whether the text supported a CID relation between the highlighted concepts. This is a true CID relation according to the gold standard (PMID 3952818).

**Figure 3.**
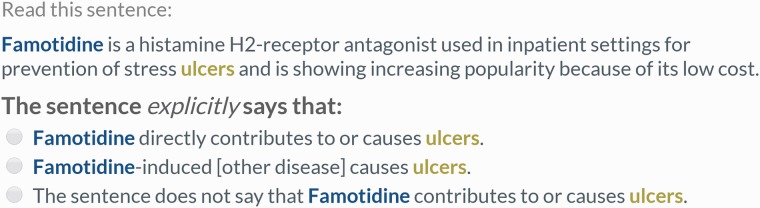
Example of a sentence-scoped relation extraction task. Workers were presented with one sentence containing all mentions of a single chemical–disease pair and asked to make a judgment about whether the text explicitly supported a CID relationship between the concepts. This is not a CID relation according to the gold standard (PMID 9669632).

Remaining pairs were divided into those which never co-occurred within any sentence, and those which co-occurred at least once within a sentence. Lingpipe was used to split abstracts into individual sentences (30). A separate crowdsourcing task was used to process the two sets of relation pairs. In both tasks, five workers were shown one relation identifier pair in the original context (either a single sentence or the full abstract) and asked to make a judgment about whether the provided text supported a CID relation between the chemical and disease. All annotations of the chemical and disease were highlighted in the text. For the abstract-level task, the judgment contained two choices, ‘true’ or ‘false’ (Figure 2). For the sentence-level task, previous testing showed that workers were falsely annotating CDRs following a ‘[chemical]-induced [intermediate disease] causes [disease]’ pattern as CID relations. Therefore, a third choice was included to capture these low frequency relations (which were treated as ‘false’ during evaluation) (Figure 3). Instructions provided to workers (Supplementary Figures S1 and S2) were designed from scratch.

### Crowdsourcing framework

We used the CrowdFlower platform (www.crowdflower.com) to gather all judgments. CrowdFlower gathers millions of judgments per month from workers in 154 countries around the world (31, 32). In order to ensure high quality results, workers for both of our tasks had to pass an initial six-question quiz. Quiz questions for each worker were randomly selected by CrowdFlower from a pool of test questions (Table 1). After passing the initial quiz, workers had to then maintain an overall minimum accuracy of at least 70% on test questions hidden in the task stream. Those who fell below this limit were automatically removed and had their judgments discarded, but were still compensated for their time. Workers were paid 2 and 4 cents, and required to spend a minimum of 3 and 10 s per sentence- and abstract-level task, respectively (Table 1).

**Table 1.** Summary of crowdsourcing task settings

Setting	Sentence-task	Abstract-task
Judgments per relation	5	5
Pay per judgment (USD)	2 cents	4 cents
Minimum time per judgment	3 s	10 s
Maximum theoretical pay	$24/h	$14.40/h
Number of tasks	2940	2766
Number of test questions	551	228
Total cost (USD)	$439.63	$851.04

### Result aggregation

Worker judgments were aggregated using a voting scheme. Any abstract-scoped relation which received four or more positive votes was judged to be a CID relation. This threshold was empirically determined using previous experiments with a subset of the BioCreative development set (which resulted in 0.587 *F*-score, 0.528 precision, 0.661 recall). Since multiple sentences from the same abstract could contain the relation pair, votes for individual sentence-scoped tasks were first tallied to determine whether each sentence supported the CID relation. Afterwards, the sentence-scoped task with the most positive votes was taken to represent whether the relation was true for that abstract. This aggregation scheme assumed that the relation was true for the entire abstract if at least one sentence supported the relation. Finally, both sentence- and abstract-scoped relations receiving four or more votes were combined with the automatically determined CID-pattern relations to generate the final predicted list of CID relations.

### Performance evaluation

Relation extraction performance was calculated by comparing the predicted set of relations against those of the gold standard. Each relation consisted of a 3-tuple of the document ID, the chemical MeSH ID, and the disease MeSH ID (26). Two relations were considered equivalent if all components were exactly equal. Relations for all abstracts were combined together into one set for comparison (micro-average).

True positives were defined as the intersection of gold standard and predicted relations. False positives were defined as the predicted relations minus the gold relations, and false negatives as the gold relations minus the predicted relations. Precision was defined as true positives divided by true positives plus false positives, and recall as true positives divided by true positives plus false negatives. F-measure was the balanced harmonic mean of precision and recall. NER performance for the competition was only calculated for diseases, and was evaluated in the same way as relations, except a 2-tuple of document ID and concept MeSH ID was used instead (26).

## Results

### Official BioCreative evaluation performance

**Figure 4.**
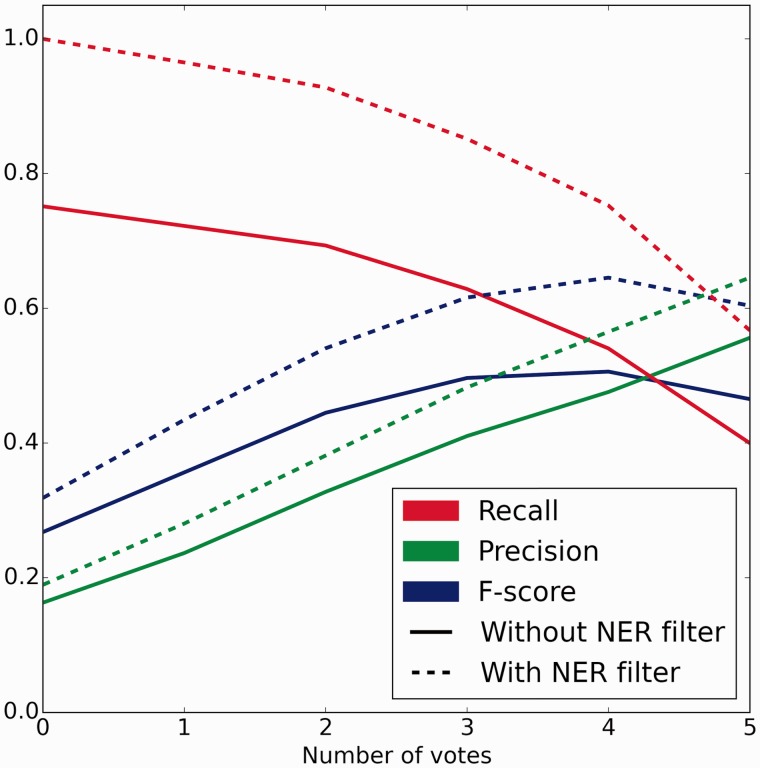
Crowd performance on evaluation test set with and without NER errors removed. Precision, recall and *F*-score are plotted as functions of the number of positive votes a relation received. Solid lines depict the performance of the full workflow from free text. Dotted lines represent the performance when relations generated using incorrect and unindexed concept annotations were removed. Peak performance of 0.505 F-score (0.475 precision, 0.540 recall) and 0.645 F-score (0.565 precision, 0.752 recall) occurred at 4 or more votes without and with NER errors removed, respectively.

The crowd completed 2940 and 2766 sentence- and abstract-level tasks for the official evaluation dataset of 500 abstracts within 6 and 7 h at a cost of $439.63 and $851.04, respectively ($2.58 per abstract overall). A total of 90 and 224 unique workers from 25 and 43 countries worked on a median of 120 and 48 sentence- and abstract-level tasks, respectively. There were 33 workers who worked on both tasks. Peak performance of 0.505 *F*-score (0.475 precision, 0.540 recall) against the gold standard occurred at a threshold of four or more votes for both sentence and abstract scoped tasks (Figure 4).

### NER performance

Due to errors in the NER process, the theoretical maximum recall for the crowdsourcing workflow was calculated to be 0.751, which occurred when all co-occurring concepts were judged as CID relations. This means that 24.9% of gold standard relations used a concept MeSH ID which was never identified in that abstract by the NER tools, and therefore unindexable due to NER loss. Examination of the NER tools revealed that tmChem’s performance for chemicals was excellent (0.9273 *F*-score, 0.9731 precision, 0.8856 recall), but DNorm's performance for diseases was substantially poorer (0.8061 *F*-score, 0.8100 precision, 0.8023 recall).

### Relation performance only

In order to better understand errors caused by the crowd’s judgments and not by NER, we applied a filter which subsetted predictions and gold standard relations to those which used concepts that had an exact match on an annotation level with the gold standard. Annotations were considered to match if the positions and MeSH IDs matched exactly. By applying this filter, any unindexable gold relations due to missing concept IDs in the predictions were removed (NER false negatives). Predicted relations generated using incorrect annotations (either identifier mismatch or location mismatch) were also removed (NER false positives).

**Figure 5.**
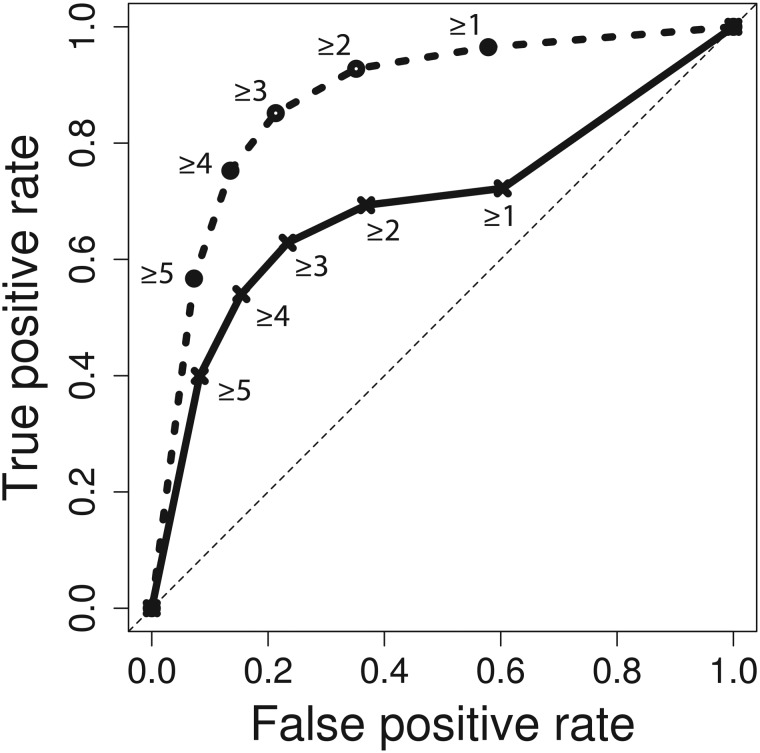
ROC curve for crowd predictions. Crowd prediction performance for the full workflow is plotted in solid lines, and performance with NER errors removed is plotted in dashed lines. Labels represent vote thresholds. Area under the curve is 0.6934 and 0.8763 for full and NER error filtered workflows, respectively.

After applying this filter to the evaluation set of 1066 relations and the crowd’s predictions, we were left with 485 gold standard relations (45.49%) from 290 different abstracts. Crowd performance on this subset also peaked at four or more of five votes on both abstract-level and sentence-level tasks, resulting in a *F*-score of 0.645 (0.565 precision, 0.752 recall) (Figure 4). Area under the receiver operating characteristic (ROC) curve increased from 0.6934 to 0.8763 when the NER error filter was applied (Figure 5). Improvement in the area under the curve is mostly due to removal of true gold standard relations which were never presented to the crowd. When generating the ROC curve, these unindexable relations were treated like relations which the crowd unanimously voted to be false (zero positive votes).

### Qualitative error analysis

In order to understand why relations were incorrectly predicted by the crowdsourcing workflow, we performed a qualitative analysis of the NER-filtered erroneous relations (Supplementary Materials). Fifteen relations from each of the four categories (sentence-scoped false positive, sentence-scoped false negative, abstract-scoped false positive, abstract-scoped false negative) were randomly selected for analysis in order to examine both crowdsourcing workflows (Table 2).

**Table 2.** Confusion matricies of crowd predictions without and with NER error-filtering

a) No NER-error filtering. Not all false negatives were generated due to unindexable relations.			
		Crowd prediction	
		TRUE	FALSE
Gold	TRUE	576 (444, 132)	490 (123, 102)
	FALSE	635 (390, 245)	3484 (1203, 2281)
b) With NER-error filtering.			
		TRUE	FALSE
Gold	TRUE	365 (290, 75)	120 (55, 65)
	FALSE	281 (181, 100)	1796 (575, 1221)

Total instances of each class are given along with the distribution of relation origin (sentence and abstract task). Sentence-scoped relations include CID-pattern relations.

Relations were reviewed by one author (T.S.L.) and classified into one of the following main error categories:

- **Gold error:** relations which were errors due to problems with the gold standard.
- **Task limitation:** relations which were judged incorrectly because of limitations and problems with the crowdsourcing workflow design.
- **Crowd wrong:** errors resulting from crowd judgment inaccuracies.

Each relation was also given a more specific error reason label (Table 3).

**Table 3.** Categories of crowdsourcing errors

Error category	Definition
Lack of comprehension	Crowd workers made incorrect judgments about the relation due to errors in judgment.
Gold missing relation	Gold standard does not include the relation, but should.
Gold false relation	Gold standard should not include the relation.
Lack of instructions	Crowdsourcing task instructions did not say how to judge this type of relation.
Lack of context	Text presented to workers was taken out of context (only applies to sentence tasks).
Hierarchy error	A relation which does not use the most specific MeSH term.

The source text, crowdsourcing tasks and gold standard annotations were examined and reviewed prior to categorizing each error. A judgment was first made as to whether the gold standard was correct according to the BioCreative biocuration guidelines (27). If there was no gold standard error, we then decided whether the crowd could have correctly judged the relation based on the crowdsourcing task and instructions they were provided. Finally, any remaining errors were classified as crowd mistakes. Any relations classified as gold standard errors were independently confirmed by a second author (B.M.G.), and any disagreements were resolved by a third author (A.I.S.).

**Figure 6.**
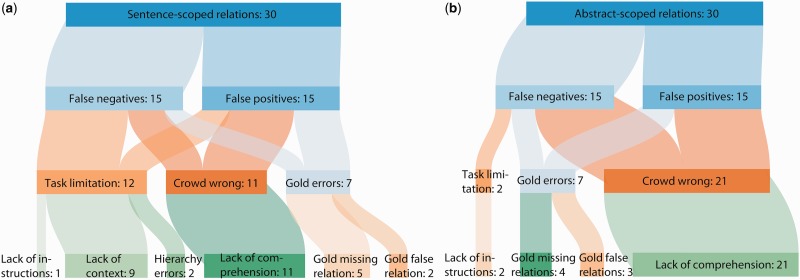
Qualitative analysis of errors after NER error filtering. A sample of 60 relations were chosen from the NER error-filtered erroneous crowd predictions and categorized according to the reason why the relation did not match the gold standard. Sentence-scoped relations **(a)** and abstract-scoped relations **(b)** were categorized separately.

Of the 60 sampled errors, a total of 14 (23.33%) relations were due to problems with the gold standard, 14 (23.33%) were due to limitations in the crowdsourcing workflow design, and the remaining 32 (53.33%) were due to poor judgments made by the workers (Figure 6). The crowd made more mistakes on abstract-scoped relations (65.62%) than sentence-scoped relations (34.37%). Gold standard mistakes were equally distributed between sentence- and abstract-scoped relations. Finally, the majority (64.28%) of task limitation issues involved showing sentences out of context.

### Crowd errors

Workers had trouble correctly understanding a substantial number of tasks. In particular, workers had difficulty correctly determining relations when highlighted chemicals were actually part of other concepts in context. In the following sentence-scoped false positive from PMID 24040781, four workers said that ‘rapamycin causes proteinuria’:

**‘Proteinuria** is an unexpected complication in transplant patients treated with mammalian target of **rapamycin** inhibitors (mTOR-i).’

In this example, workers likely equated ‘rapamycin’ with ‘mammalian target of rapamycin inhibitors’, leading them to vote that this relation is true. Although ‘rapamycin’ is indeed a chemical, we consider this an example of incomplete highlighting, since ideally the more complete ‘mammalian target of rapamycin inhibitors’ would have been highlighted instead.

Workers also had difficulty identifying the main subject of some sentences, resulting in erroneous relations. This often occurred when workers needed to differentiate between different chemicals or between chemicals and chemical classes. All workers judged that ‘organophosphate causes lung cancer’ when shown the following sentence (PMID 25907210):

‘OBJECTIVE: Diazinon, a common **organophosphate** insecticide with genotoxic properties, was previously associated with **lung cancer** …, but few other epidemiological studies have examined diazinon-associated cancer risk.’

Workers failed to identify that ‘diazinon’ was the main subject, resulting in the false positive relation.

### Workflow limitations

Showing workers individual sentences without the rest of the abstract for context was the main task limitation (nine relations, 64.28%). Our simplifying assumption that the sentence containing two co-occurring concepts would alone be sufficient to judge the relation did not always hold. This led workers to make judgments with incomplete information, which commonly resulted in false negative errors.

For instance in PMID 3191389, the crowd missed the relation ‘phenylbutazone causes seizures’, because the only sentence they were shown was

‘The present study was designed to investigate the effect of 5 non-steroidal anti-inflammatory drugs, …, **phenylbutazone**, …, on **seizures** produced by pilocarpine.’

Workers needed to see the following sentence,

‘Pretreatment of rats with sodium salicylate, …, and **phenylbutazone**, …, converted the non-convulsant dose of pilocarpine, …, to a convulsant one.’

in order to correctly judge that phenylbutazone exacerbated the seizure-inducing properties of pilocarpine. Sentences most likely to follow this structure were titles and research objective declarations, where the intent to study or examine an effect was stated, but the outcome was not.

### Gold standard errors

Of the 60 sampled relations, 9 (64.28%) gold standard errors resulted from relations that should have been in the gold standard, and 5 (35.71%) were relations which were incorrectly included in the gold standard. Relations missing in the gold standard, which were treated as false positives, often were included in long lists of symptoms. For instance, relations linking hand–foot syndrome with doxorubicin and carboplatin were identified by the crowd but missing from the gold standard for PMID 11745287. The abstract states:

‘The combination of **carboplatin** … and liposomal **doxorubicin** … was administered … to patients with recurrent squamous cell cervical carcinoma to determine antitumor activity …’

Later in the results section, it states:

‘Grade > or = 2 nonhematologic toxicity included nausea in 17 patients, …, constipation in 6 patients, …, **hand-foot syndrome** in 2 patients, and skin reactions in 3 patients.’

The gold standard contains relations between carboplatin and doxorubicin for nausea, constipation and skin reactions, but omits hand–foot syndrome. In these authors' view, these links are true relations that are missing in the gold standard.

Some gold standard relations seem to have been incorrectly added. For example, in PMID 9578276, the gold standard links carbetocin with vomiting and abdominal pain. The abstract states:

‘To determine the maximum tolerated dose of **carbetocin** … when administered immediately after vaginal delivery at term.’

Later in the results section it states:

‘Recorded were dose-limiting adverse events: hyper- or hypotension (three), severe **abdominal pain** (0), **vomiting** (0), and retained placenta (four).’

The gold contains relations between carbetocin and all five adverse events, but the text states that zero patients suffered vomiting or severe abdominal pain. This seems to be a biocurator mistake.

### Comparison against two machine learning systems

In order to better understand the strengths and weaknesses of the crowd's performance, we compared crowd-predicted relations against predictions from two machine learning systems which also participated in the BioCreative V challenge: BeFree (33, 34) and UTH-CCB (UTexas, (35)). The UTexas system ranked first in the BioCreative evaluation with a *F*-score of 0.5703 (0.5567 precision, 0.5844 recall) (26, 35).

**Figure 7.**
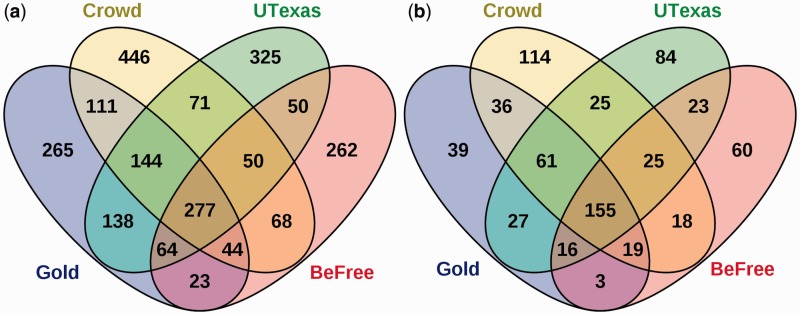
Comparison of CID predictions by crowdsourcing and two machine learning systems against the gold standard. **(a)** The predictions of the crowd, BeFree, and UTexas systems using full workflows compared with the gold standard. Overlaps represent relations common to both sets. (**b)** Overlap of predicted relations after applying the NER error filter.

The predicted relations of each method were strikingly different. For the full workflows, each method had at least two hundred unique predicted relations (Figure 7a). A core subset of 277 gold relations (25.98%) was identified by all three systems. These relations likely represent clear, unambiguous relations, as 255 (92.05%) were sentence-scoped. Strikingly, 265 (24.86%) of the gold standard relations could not be predicted by any of the systems.

To again focus specifically on the relation extraction task, we also performed the comparison analysis using an NER error filter that focused on relations involving concepts which were perfectly annotated by all three methods. This filter ensures a fair comparison between methods. Post-filtering, the original set of 1066 gold standard relations from 500 abstracts was reduced to 356 relations from 224 abstracts, representing 33.39% of the original dataset. The proportion of gold relations not predicted by any system drops to 39 relations (10.95%), indicating that most of the relations unpredicted in the full workflow were affected by NER problems (Figure 7b). The core set of predicted relations common to all three methods increased to 155 (43.53%), indicating that relation consensus is higher when the methods all agreed upon the annotations and identifier mappings.

We manually reviewed three sets of relations from the NER-error filtered predictions: relations that the crowd correctly predicted but machines did not (36), relations that machines correctly predicted but the crowd did not (16) and relations all three methods predicted but did not match the gold (25). In the set of 36 relations which the machines were not able to predict, 61.1% were abstract-scoped, suggesting that it is more difficult for the machines to infer relations spanning multiple sentences. In addition, most of these relations used conjugated chemical or disease terms (concepts joined together with ‘and’ or linked by parallelism), suggesting that it may be more difficult for the algorithms to correctly determine which concepts are relevant when there are many joined together.

For the 16 relations which the crowd missed but both machine learning algorithms predicted, we found that the crowd made judgment errors in 9 (56.25%) relations. However, 3 (18.75%) of the missed relations were due to sentence tasks taking the original text out of context, and 4 (25%) of the relations were judged to be errors with the gold standard (Supplementary Materials). After accounting for NER errors, very few relations predicted by both algorithms were missed by the crowd due to errors in judgment.

Finally, in the set of 25 false positives that all three methods predicted, 17 relations were judged to be errors with the gold standard (Supplementary Materials). Most of the disagreements with the gold standard arose as a result of unclear guidelines, which did not clarify specifics like whether relations provided as background information should be included. Another main source of error arose due to disagreements as to what the most specific disease in an article was. The guidelines said to use the most specific disease when possible, but did not discuss how this was to be determined.

## Discussion

The goal of this research is to increase the scalability of processes for extracting semantic relations from text using crowdsourcing, thereby improving the efficiency of expert human curation. If such a workflow can be achieved it could be used to rapidly create large ground truth corpora for training supervised machine learning systems and could also be applied directly to biocuration tasks. In comparison to the gold standard data provided in the BioCreative challenge, the crowdsourcing workflow presented here achieved an *F*-score of 0.505 (0.475 precision, 0.540 recall), which we would not classify as expert-level performance. However, the system was fast (processing ∼75 abstracts/hour), inexpensive ($2.58/abstract), and relatively accurate in comparison to most fully automated approaches. Different judgment aggregation schemes may result in higher performance (36). Although it did not exactly reproduce the gold standard dataset, the system did generate data of sufficient quality to train a machine learning system that yielded equivalent accuracy to the same system trained on expert-generated data (34). Looking forward, analysis of the errors made on the gold standard test data revealed many areas where it could be improved.

### NER impact

Overall, the biggest observed impact on performance with our current workflow was the NER step. Almost a quarter of all gold standard relations used MeSH IDs which could not be correctly determined by tmChem and DNorm, and were counted as false negatives without any crowd judgment. After NER error filtering, performance improved substantially to 0.645 *F*-score, suggesting that actual worker judgment may be better when concepts are correctly highlighted. We also saw that incomplete concept highlighting could influence worker performance, despite being judged as correct annotations according to the gold. This suggests that NER systems may need to be tuned for downstream use cases in order to provide optimal results. NER performance for diseases was significantly worse than for chemicals, and represents the area where the biggest improvements can be made.

### Workflow limitations

The workflow showed workers single sentences in order to simplify the relation verification tasks. Sentence-level tasks were completed at a faster rate than abstract-level tasks and attracted workers who performed more tasks. Although this worked well, there were instances where workers were presented with insufficient information to make a correct judgment regarding the relation. In a subsequent preliminary experiment with tasks that always showed the full abstract, we found that the performance was not significantly different, and that workers rated the task as being more difficult (data not shown). Cost per abstract also increased, since a greater fraction of workers fell below the minimum acceptable accuracy limit, and had their judgments invalidated. Rejected workers were however still paid for their judgments.

Although proper context is necessary to correctly judge some relations, more context alone is likely insufficient to improve worker accuracy due to the corresponding increase in task complexity. Because workers were more likely to make errors on abstract-level tasks, any gains from judging relations in context were likely offset by losses resulting from more mistakes. Although further experiments examining the tradeoff between task complexity and performance accuracy are required, we concluded that for this dataset the increase in task simplicity gained by using single-sentence tasks was worth sacrificing accuracy on the small fraction of relations which were judged out of context.

### Lack of detailed task guidelines

Many of the disagreements between the crowd’s predictions and the gold standard may be attributed to a lack of detailed information in the task guidelines provided by the BioCreative challenge organizers to task participants (27). Although six pages were dedicated to describing which concepts should be annotated, only three remarks were made regarding the relations:

- The annotated relationship includes primarily mechanistic relationships between a chemical and disease. Occasional biomarker relations are also included (e.g. relation between D006719 (Homovanillic Acid) and D006816 (Huntington Disease) in PMID:6453208).
- The relation should be explicitly mentioned in the abstract.
- Use the most specific disease in a relationship pair.

The BioCreative task guidelines state that more details can be found in (37) and (38), but the official curation manual is ‘proprietary’, and the ‘specialized pharma-edition of *CTD’s Curation Manual*’ specially produced for this dataset was also publically unavailable. Although we tried our best to create comprehensive instructions for workers, we found many edge cases which were not covered in the BioCreative task guidelines. These edge cases resulted in many false positive predictions because we did not tell the crowd how to categorize these cases. Since other established work has shown that crowd workers can perform with high accuracy when the original curation guidelines are available, we believe that the high false positive rate is mainly the product of a lack of detailed relation curation guidelines (24).

### Related work

Our crowdsourcing workflow uses a similar combination of automated NER and crowd relation verification as previously published methods. This machine-assisted crowdsourcing approach was also used by Burger *et al.* (23) and Khare *et al.* (24) to extract gene-mutation and drug-disease relations respectively. Both of these methods pre-populated entity annotations with automated NER tools and generated all possible relation pairs for workers to verify. All tasks asked workers to verify one relation in the full original context. However, neither method attempted to divide tasks into different workflows based on sentence-cooccurrence. For aggregation, while Burger *et al.* saw a substantial improvement in accuracy by using a Bayesian aggregation method, Khare *et al.* saw no performance gain when they used an expectation maximization algorithm to aggregate worker judgments. In their case, simple majority voting actually performed better.

## Conclusion

We applied a crowdsourcing workflow to extract CID relations from PubMed abstracts as part of the BioCreative V challenge, and ranked fifth out of 18 participating teams (26). We were the only crowdsourcing entry to the BioCreative V CDR task, and to the best of our knowledge, this is the first application of a crowdsourcing component in a workflow submitted to a biomedical community challenge. The largest source of errors for the crowdsourcing workflow was actually the automated NER that initiated the process, which accounted for nearly 25% of all errors. Although we did not have the highest performance in terms of *F*-score, our crowd-based method was capable of detecting mistakes in the gold standard and worked well on some abstract–bound relations. Our error analysis revealed that some of the assumptions used to simplify the task did not always hold, and that limitations in the task design were responsible for some incorrect predictions. Like machine learning methods, our current crowdsourcing method will benefit from additional iterative rounds of refinement. However, our current design already performs better than the majority of automated methods, which gives us confidence that aggregate crowd workers can be complementary to trained biocurators.

## Supplementary data

Supplementary data are available at Database Online.

Supplementary Data
