# Supplementary material for: A crowdsourcing workflow for extracting chemical-induced disease relations from free text
Source: Database (Oxford). 2016 Apr 16;2016:baw051. doi: 10.1093/database/baw051 (PMC4834205; doi:10.1093/database/baw051)
Supplement: Supplementary Data [file supp_2016_baw051_index.html]

Supplementary Data 

# A crowdsourcing workflow for extracting chemical-induced disease relations from free text

## Supplementary Data

files

- Supplementary Data - docx file
