# Supplementary material for: A crowdsourcing workflow for extracting chemical-induced disease relations from free text
Source: Database (Oxford). 2016 Apr 16;2016:baw051. doi: 10.1093/database/baw051 (PMC4834205; doi:10.1093/database/baw051)
Supplement: Supplementary Data [file supp_baw051_Supplementary_Information.docx]

**Supplementary data for *A crowdsourcing workflow for extracting chemical-induced disease relations from free text***

## **1. Acronym Identifier Resolution**

Using the default parameters for tmChem and DNorm to perform named entity recognition would sometimes produce concept annotations which were labeled as chemicals or diseases but which did not include a MeSH identifier. For example, in PMID 23871786, “BPA” was annotated by tmChem and a chemical, but no MeSH ID was assigned. For these identified but unlabeled concepts, we tried to assign MeSH IDs using the following procedure:

1. Find concepts with MeSH ids (the “definition” concept) which are followed by another annotation in parentheses (the “abbreviation” concept)
2. Label all subsequent annotations without MeSH ids matching the text of the abbreviation concept with the MeSH ID of the definition concept

With this acronym resolution heuristic applied, the NER performance for tmChem on a strict annotation level (position and MeSH ID must match the gold standard) for the testset increased from 0.8144 F-score (0.8412 precision, 0.7892 recall) to 0.8546 F-score (0.8828 precision, 0.8282 recall). The acronym resolution heuristic made no difference for diseases. Performance for diseases was a constant 0.7410 F-score (0.7537 precision, 0.7287 recall) at the strict annotation level.

## **2. Instructions to CrowdFlower Workers**

All instructions presented to workers who attempted and worked on our crowdsourcing tasks on the CrowdFlower platform are given in supplementary figures 1 and 2. Workers were able to toggle displaying the instructions by clicking on a button.

Figure S1: Instructions provided to workers of the sentence-scoped CID relation verification task.


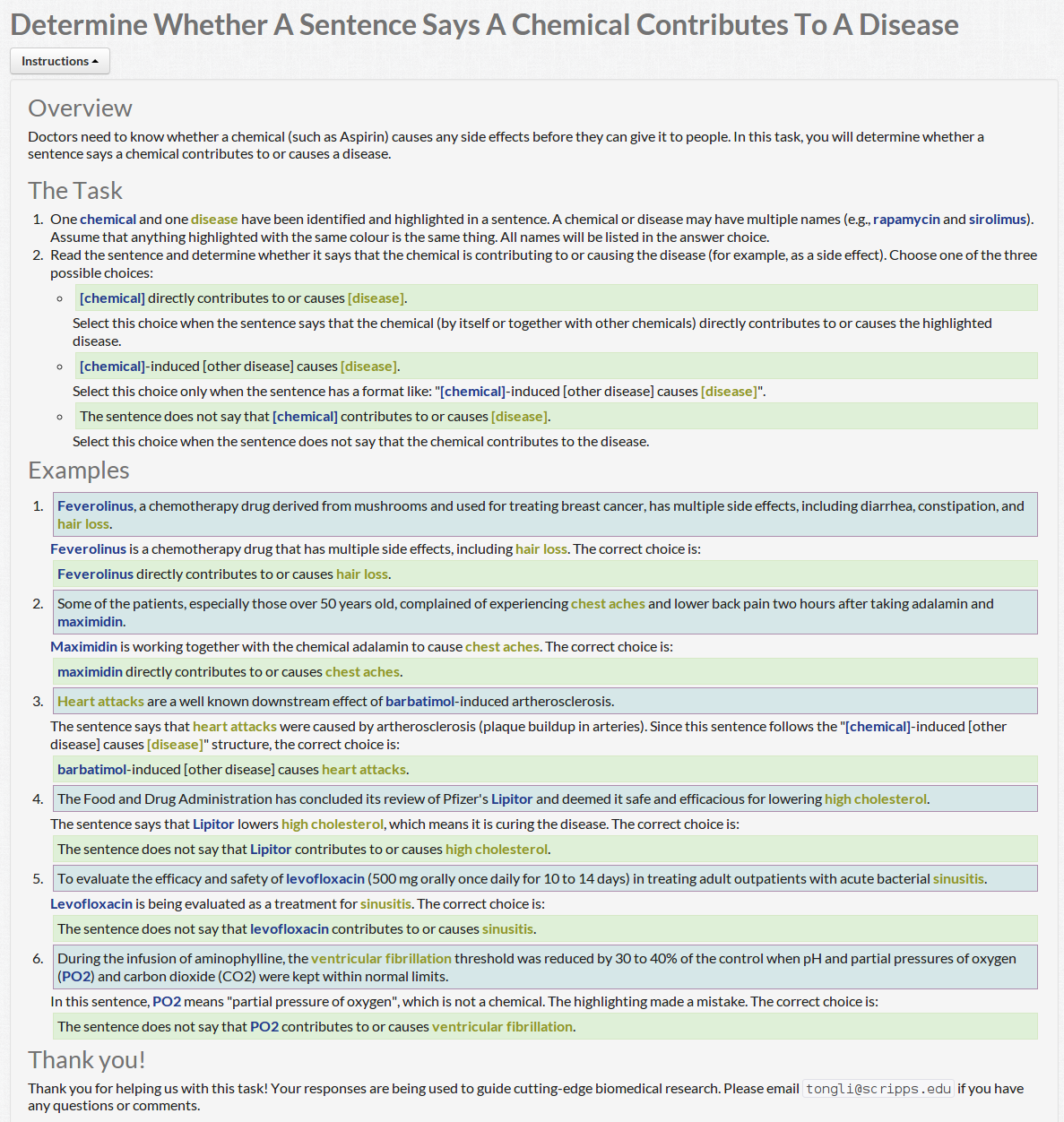


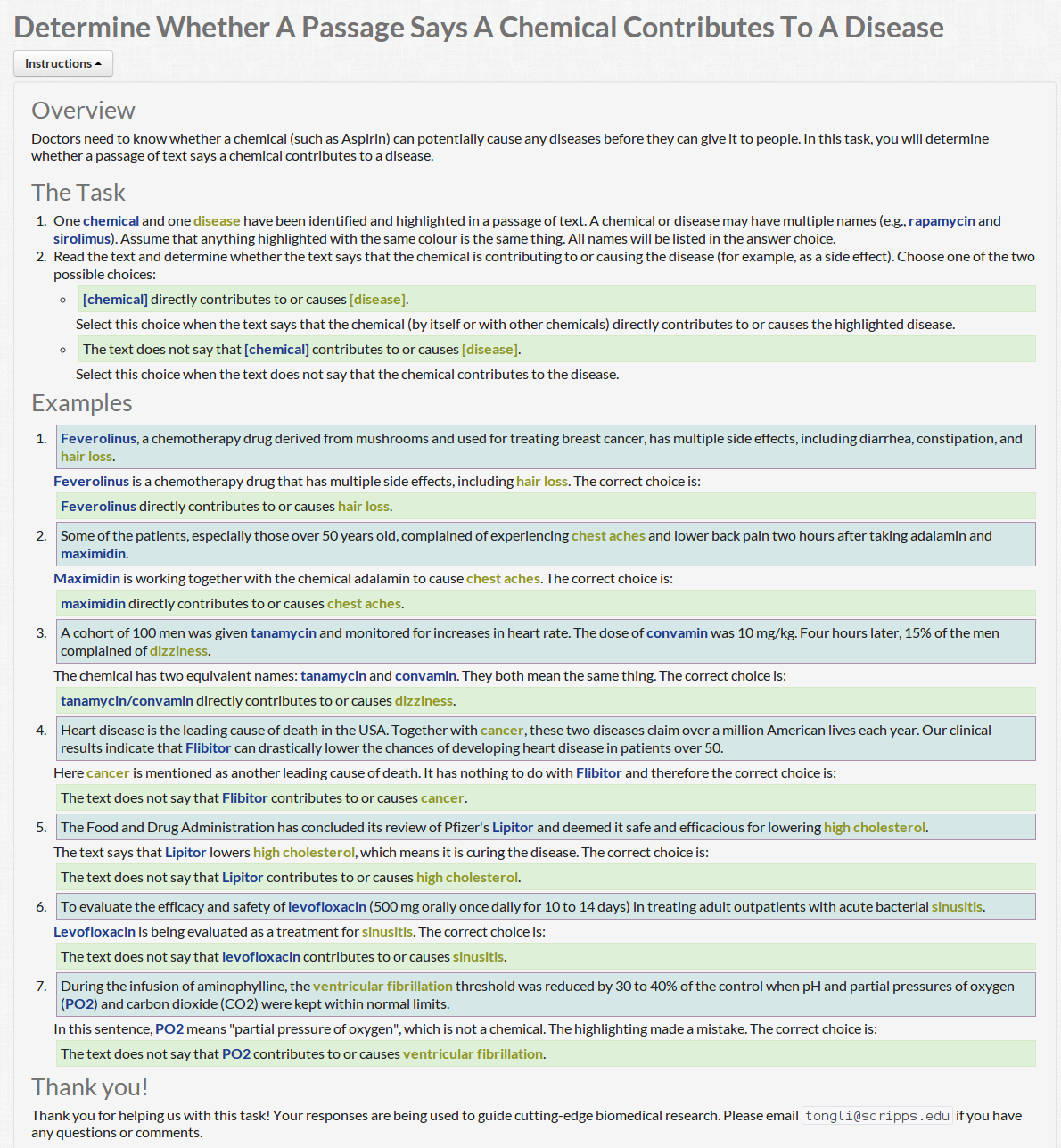


Figure S2: Instructions provided to workers of the abstract-scoped CID relation verification task.

## **3. Error Analysis Sampling**

The following data files are included as part of the error analysis:

testset_crowd_perfect_ner_errors_final.xlsx: Contains manual error analysis of errors generated by the crowdsourcing method only.

4_way_venn_data.xlsx: Contains all relations predicted by each of the three methods as well as the gold standard, cross referenced for easy computation.

testset_all_predictions_agree_false_positives_with_ner_filter_final.xlsx: Contains manual error analysis of relations agreed upon by all three predictive systems but which were considered false positives according to the gold standard.

testset_crowd_gold_no_machines_with_ner_filter_final.xlsx: Contains manual error analysis of relations which only the crowd managed to extract.

testset_machine_gold_no_crowd_with_ner_filter_final.xlsx: Contains manual error analysis of relations correctly predicted by the two machine learning methods but which was missed by the crowd.
